# Clinical effect of conbercept on improving diabetic macular ischemia by OCT angiography

**DOI:** 10.1186/s12886-020-01648-x

**Published:** 2020-09-25

**Authors:** Ziyi Zhu, Youling Liang, Bin Yan, Zhishang Meng, Kejun Long, Yiwei Zhang, Jing Luo

**Affiliations:** 1 Department of Ophthalmology, The Second Xiangya Hospital, Central South University, 139 Middle Renmin Rd, Changsha, Hunan 410011 People’s Republic of China; 2 Hunan Clinical Research Center of Ophthalmic Disease, Changsha, Hunan 410011 People’s Republic of China

**Keywords:** Diabetic retinopathy, Macular ischemia, Conbercept, OCT angiography

## Abstract

**Background:**

Varying degrees of macular ischemia generally occur in diabetic retinopathy (DR). This study aims to evaluate the effect of conbercept with 3+ pro re nata (PRN) on macular perfusion status in patients with diabetic macular edema (DME) and quantitatively assess changes in foveal avascular zone (FAZ) areas and capillary density in macular regions by applying optical coherence tomography angiography (OCTA).

**Methods:**

Fifty patients were divided into ischemic (*n* = 31) and non-ischemic (*n* = 19) groups according to the presence of ischemia on OCTA at baseline. All patients received intravitreal injections of 0.5 mg of conbercept with 3+ PRN principle. The FAZ areas and macular vessel density measured using OCTA were evaluated at baseline, 3 months, and 6 months after treatment in both groups.

**Results:**

At months 3 and 6, the FAZ area in the ischemic group changed from 0.510 ± 0.171 mm^2^ to 0.441 ± 0.158 mm^2^ then to 0.427 ± 0.153 mm^2^ (*p* = 0.003, *p* = 0.296); in the non-ischemic group, it remained stable (*p* = 0.269, *p* = 0.926). The superficial vessel density changed from 41.1 ± 4.1 to 42.5% ± 4.7% then to 42.6% ± 4.6% (*p* = 0.043, *p* = 0.812), and the deep vessel density changed from 40.7 ± 4.4 to 42.3% ± 3.6% then to 42.3% ± 4.7% (*p* = 0.072, *p* = 0.961) in the ischemic group. In the non-ischemic group, the superficial vessel density changed from 44.8 ± 3.2 to 46.0% ± 3.5% then to 45.7% ± 3.3% (*p* = 0.108, *p* = 0.666), whereas the deep vessel density changed from 43.6 ± 3.6 to 43.8% ± 3.2% then to 43.5% ± 4.5% (*p* = 0.882, *p* = 0.736). Reperfusion in macular nonperfusion areas was observed.

**Conclusion:**

Anti–vascular endothelial growth factor treatment may have a positive effect on macular perfusion status. Furthermore, OCTA had advantages in quantifying and calculating blood flow index in the study of macular perfusion status.

## Background

As extensively reported, the further the diabetic retinopathy (DR) progresses, the severer the condition of diabetic macular ischemia (DMI) in patients with DR [1, 2]. In macular ischemia, the structure of foveal capillary network is damaged. Such damage includes enlargement and irregularity of the foveal avascular zone (FAZ) and appearance of macular nonperfusion (MNP), leading to macular function disturbance. According to ETDRS Report No. 11, the macular ischemia is defined as a FAZ area enlarging more than 1000 μm in greatest diameter (supposing the FAZ is round or oval), equaling to the size of FAZ more than the area within 500 μm radius circle, and/or broken perifoveal capillary rings at the borders of FAZ with areas of macular capillary nonperfusion within the a1 disk diameter of the foveal center according to fluorescein angiography (FA) [3]. In healthy people, the diameter of normal FAZ commonly varies from 500 μm to 600 μm (less than the area within 300 μm radius circle equaling to 0.282mm^2^) [3]. When a macular region is affected by ischemia, it presents in varying degrees, including disappearance of a part of the macular arch ring capillary network, expansion of the FAZ area, damage in perifoveal capillaries, and appearance of MNP area in the macular region. Different levels of macular ischemia can represent disease severity and progression; for instance, moderate-to-severe macular ischemia affects visual function seriously. Macular ischemia results from the occlusion of foveal capillary network, and vascular endothelial growth factor (VEGF) plays a vital role in the mechanism by which VEGF leads the closure of retinal vascular in patients with diabetic macular edema (DME) [4].

Several previous large studies regarding the treatment effects of anti-VEGF on DME, including DRCR studies, did not include macular ischemia as an observation indicator. To date, the insight into whether anti-VEGF therapy could aggravate retinal ischemia remains controversial. In the past decade, some clinical studies suggested that blocking VEGF might be harmful to retinal vascular integrity, especially in patients with preexisting retinal ischemia; in these patients, anti-VEGF therapy aggravated retinal ischemia because retinal nonperfusion (RNP) areas enlarged after anti-VEGF therapy [5, 6]. However, increasing studies have shown that retinal ischemia does not worsen after anti-VEFG therapy but has no retinal reperfusion in the preexisted RNP areas [7–9]. After anti-VEGF treatment, the proportion of patients with ≥2-step DR severity score improvement is greater in patients with MNP at baseline [10]. Meanwhile, few studies indicated that anti-VEGF therapy could reduce RNP progression. In a multicenter clinical trial, monthly injection of 0.3 or 0.5 mg of ranibizumab can slow retinal vessel closure in DME and can be associated with retinal reperfusion at the anterior nonperfusion area in some patients [11]. Long-term anti-VEGF therapy to patients with DME could improve DR severity and even prevent worsening, especially in mild and severe DR cases. Furthermore, early treatment may prevent visual damage caused by proliferative DR (PDR) and obtain better therapeutic effects [12]. Thus, parts of researchers think anti-VEGF therapy not only improves patients’ vision but also reduces RNP progression, and even enhances blood flow [11–13].

To date, most of the studies about anti-VEGF effects on DMI were small cross-sectional studies or retrospective studies, and they lack supporting prospective research. Moreover, most studies often use FA to observe macular ischemia changes; however, this tool is invasive and poorly repeatable. Meanwhile, optical coherence tomography angiography (OCTA) is noninvasive, repeatable, and easily operable, and it is subtler than FA in displaying macular area capillaries. OCTA acquires high-resolution images of macular areas by capturing the signals of moving red blood cells and provides images of the construction of macular capillaries at different plexus of the retina. In addition, OCTA is highly sensitive and consistent compared with FA [14–16]. However, numerous studies only focus on the retinal ischemia status, and more detailed effects on macular ischemia after anti-VEGF therapy have not been studied clearly. Thus, we decided to adopt OCTA as the main evaluation method to observe the blood flow of macular areas and quantify the density of macular vessels.

In this prospective study, we mainly aim to investigate the effects of conbercept with 3+ PRN on macular perfusion status in patients with DME and to quantify FAZ areas and capillary density in the macular region. Conbercept (Langmu; Kanghong, Inc., Sichuan, China) is a new drug comprising a VEGF receptor (VEGFR) fusion protein, with a high binding affinity to VEGF and a long half-life in vitreous, and it has been proven to reduce the chances of intraoperative bleeding in vitrectomy procedures by decreasing VEGF concentrations. Conbercept demonstrates a higher binding activity with VEGF-A, VEGF-B, and placental growth factor (PIGF) than ranibizumab. Clinical trials indicate that intravitreal injections of conbercept can improve visual acuity in patients receiving vitrectomy and seems to reduce the recurrence rate of vitreous hemorrhage in patients with PDR [17].

## Methods

The study followed the tenets of the Declaration of Helsinki, all patients gave informed consent before participation in this study and all procedures performed were in accordance with the ethical standards of the Second Xiangya Hospital of Central South University committee. In this study, 50 patients (obtained after informed consent), who consulted in the abovementioned hospital for diminution of vision caused by diabetes between September 2018 and March 2019, were diagnosed of DR with different levels of DME [18]. And only one eye of each patient was included in the study. Inclusion criteria were as follows: patients > 18 years old with type 1 or 2 diabetes; and definite retinal thickening due to DME as the main cause of visual loss (central fovea thickness [CFT] ≥250 μm measured on OCT). Meanwhile, the exclusion criteria were the following: visual loss caused by any other retinal disease, excluding DME; retinal treatment or major ocular surgery within the prior 6 months; intraocular pressure ≥ 25 mmHg; refractive error (myopia >6D or hyperopia >3D), severe refractive media, and other systemic diseases that needed hospitalization. All of them were injected with 0.5 mg of conbercept at monthly intervals for the first 3 months and given additional interval injections according to the pro re nata (PRN) principle [19]. All of these patients were evaluated for 6 months, comprising a 3-month treatment period (received monthly interval injection of 0.5 mg of conbercept) and a 3-month observation period (received treatment according to the PRN principle). Before the first intraocular injection, all patients underwent FA, OCT, and OCTA, which showed whether a patient had apparent macular ischemia (including FAZ area expansion, perifoveal capillary ring damage, and/or MNP appearance). And we excluded patients with poor OCTA image quality before grouping patients: Q-score below 7; opacity of refractive media; presence of significant residual motion artifacts; anatomical features of macular area severely disrupted (such as severe cystoid macular edema) leading to segmentation errors. Then, the patients were divided into two groups, namely, the ischemic group and the non-ischemic group. Considering the lack of uniform definition of macular ischemia classification according OCTA, we adopted the basis of grouping at baseline according to macular ischemic grading defined by FA [3]. Ischemic group is defined as the presence of one or more of the following criteria: 1.FAZ area is greater than or equal to 1000 μm diameter circle (0.785 mm2); 2. Outline of FAZ is not a smoothly regular round or oval, the capillary outline is lost or destroyed; 3. Appearance of retinal capillary loss even MNPs in macular region. The rest is defined as non-ischemic group.

### Image acquisition

All OCT and OCT angiography images were acquired from AngioVue OCT system version 2018.0.0.14 (RTVue XR Avanti, Optovue Inc., Fremont, CA, USA.) using split-spectrum amplitude decorrelation angiography (SSADA) algorithm to detect blood flow and provide a new for rapid imaging of detailed microvasculature at distinct depths [15]. The system reduced artifacts by motion correction technology (MCT) and 3D projection artifact removal (PAR) remove artifacts, and PAR differentiated situ OCTA signal from projection artifact and removed the projection artifacts [20, 21]. The scanning area of OCTA was uniformly obtained in 6 × 6 mm^2^ sections consisting of 304 B-scans. Every B-scan was repeated twice, as well as 304 A-scans. In 3 s, 209000 A-scans were obtained. AngioVue provided an automated software algorithm to generate the boundaries of superficial capillary plexus (from ILM to 10 μm above IPL), deep capillary plexus (from 10 μm above IPL to 10 μm below OPL) and choriocapillaris. And the boundary of FAZ area was from ILM to 10 μm below OPL. While the segmentation boundaries also could be adjusted and corrected by manually segmented [22].

### Data measurement

We assessed best corrected visual acuity (BCVA) by using a tumbling E chart at an initial testing distance of 5 m, and the result was displayed in a logMAR unit format [23]. Then, we calculated the CFT as an average value within a circular 1 mm diameter area centered in the fovea measured by OCT. We also obtained vessel density value by calculating the percentage of blood vessel area in a 6 × 6 mm^2^ selected area from the OCTA. Furthermore, FAZ area was calculated using an automated software but could be corrected by manual selection.

### Statistical analysis

Main measurements, including BCVA, FAZ area, CFT, and superficial and deep vessel density, in this study were tested by Kolmogorov–Smirnov test (*p* > 0.05 in all samples). Hence, we adopted *t* test principally. At months 0, 3, and 6, changes in Q-score, CFT, FAZ area, and blood flow were evaluated by paired-sample *t* test. Moreover, *p* < 0.05 indicated statistical significance. Statistical analysis was performed using the SPSS 25.0 (SPSS Inc., Chicago, III, USA).

## Results

### Characteristics of patients with DME at baseline

This study enrolled 50 patients with DME and divided them into two groups, namely, the ischemic group (*n* = 31) and the non-ischemic group (*n* = 19). The characteristics of patients at baseline are shown in Table 1. These patients comprised 24 males and 26 females, and the mean age was 55.8 years (standard deviation [SD]: 8.4 years [34–80 years]). The BCVA was 0.61 ± 0.34 (mean ± SD). The mean duration of diabetes was 9.4 years, and the HbA1c was 9.6% ± 2.0%. Among these 50 patients, 7 had moderate NPDR (14%), 34 had severe NPDR (68%), and 9 had PDR (18%). Among the 9 patients with DR, 9 had hypertension, 4 had nephropathy, and 1 had thrombocytopenia, showing that DR is associated with many other systemic disorders, especially circulatory diseases and nephropathy. The overall main characteristics of patients with DME were increased central fovea thickness (367 ± 122 μm), increased FAZ area (0.455 ± 0.171 mm^2^), and deceased vessel density (vessel density in superficial plexus [ILM-OPL] and deep plexus (IPL-OPL) was 42.5 ± 4.2 and 41.8% ± 4.3%, respectively).

**Table 1:** Basic characteristics of patients at baseline (*n* = 50)

	Ischemic Group (*n* = 31)	Non-ischemic Group (*n* = 19)	Total (*n* = 50)
Gender, n			
Male	17 (55%)	7 (49%)	24 (48%)
Female	14 (45%)	12 (51%)	26 (52%)
Age, years	55.8 ± 8.5	55.8 ± 8.4	55.8 ± 8.4
BCVA, logMAR units	0.64 ± 0.34	0.56 ± 0.34	0.61 ± 0.34
Duration of Diabetes, years	10.1 ± 4.5	8.2 ± 4.9	9.4 ± 4.7
Stage of DR, n			
Mild NPDR	0 (0)	0 (0)	0 (0)
Moderate NPDR	4 (13%)	3 (16%)	7 (14%)
Severe NPDR	21 (68%)	13 (68%)	34 (68%)
PDR	6 (19%)	3 (16%)	9 (18%)
GHb,%	9.6 ± 2.1	9.7 ± 2.0	9.6 ± 2.0
Complication			
Hypertension	7	2	9
Renal Failure	2	2	4
Others	0	1	1
CFT(1 mm), μm	328 ± 109	430 ± 118	367 ± 122
FAZ area, mm2	0.510 ± 0.171	0.364 ± 0.127	0.455 ± 0.171
Vessel Density,%			
Superficial (ILM-IPL)	41.1 ± 4.1	44.8 ± 3.2	42.5 ± 4.2
Deep (IPL-OPL)	40.7 ± 4.4	44.8 ± 3.2	41.8 ± 4.3

*NPDR* non-proliferative diabetic retinopathy, *PDR* proliferative diabetic retinopathy, *GHb* glycated hemoglobin

The composition of sex and age, BCVA, diabetes duration, and GHb between the ischemic group and non-ischemic group had no significant difference (*p* > 0.05, chi-square test and independent-sample *t* test). Conversely, the CFT, FAZ area, and vessel density between the two groups were significantly different. In the ischemic group, the CFT (328 ± 109 μm) was significantly lower than that in the non-ischemic group (*p* = 0.005). The FAZ area in the ischemic group (0.510 ± 0.171 mm^2^) was preoperatively larger than that in the ischemic group (0.364 ± 0.127 mm^2^, *p* = 0.001). Additionally, the superficial and deep vessel densities in the ischemic groups were 41.1 ± 4.1 and 40.7% ± 4.4%, respectively; both were clearly low than those in the non-ischemic group (44.8% ± 3.2%, *p* = 0.001 and 44.8 ± 3.2%, *p* = 0.015). Overall, the patients in the ischemic group showed ischemic changes such as FAZ area expansion, MNP appearance, and blood flow density decrement.

### Anti-VEGF therapy decreased the FAZ area

**Fig. 1:**
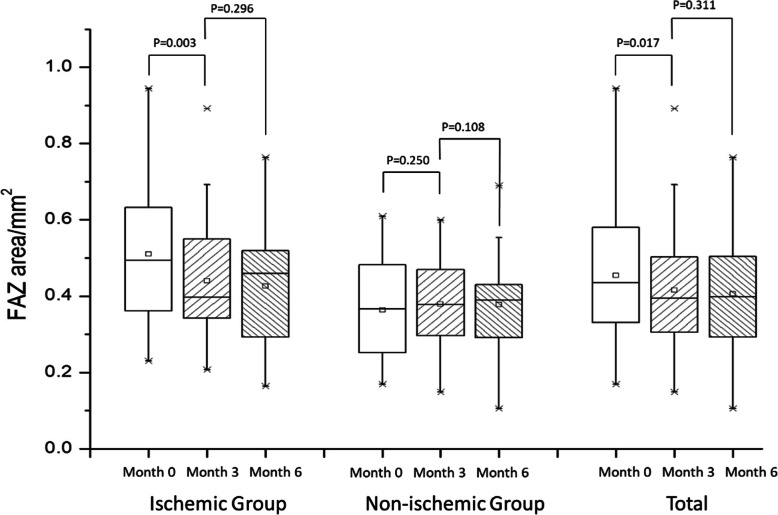
Changes in FAZ area after anti-VEFG therapy

All patients were intraocularly injected with 0.5 mg of conbercept according to the 3+ PRN principle. The main observation point was set up at months 0, 3, and 6. Additionally, we analyzed comparing the Q-score (paired-sample *t* test) for each time point to estimate whether Q-score affected the results in our study. There had no significant difference of Q-score among different time points. In the ischemic group, Q-score was 7.74 ± 0.82 at baseline, 7.90 ± 1.03 (*p* = 0.476) at month 3 and 8.07 ± 0.92 (*p* = 0.587) at month 6. And in the non-ischemic group, Q-score was 7.79 ± 0.79 at baseline, 7.74 ± 0.65 (*p* = 0.790) at month 3 and 8.00 ± 1.00 (*p* = 0.350) at month 6. The changes in the FAZ area in both groups are depicted in Fig. 1.

**Fig. 2:**
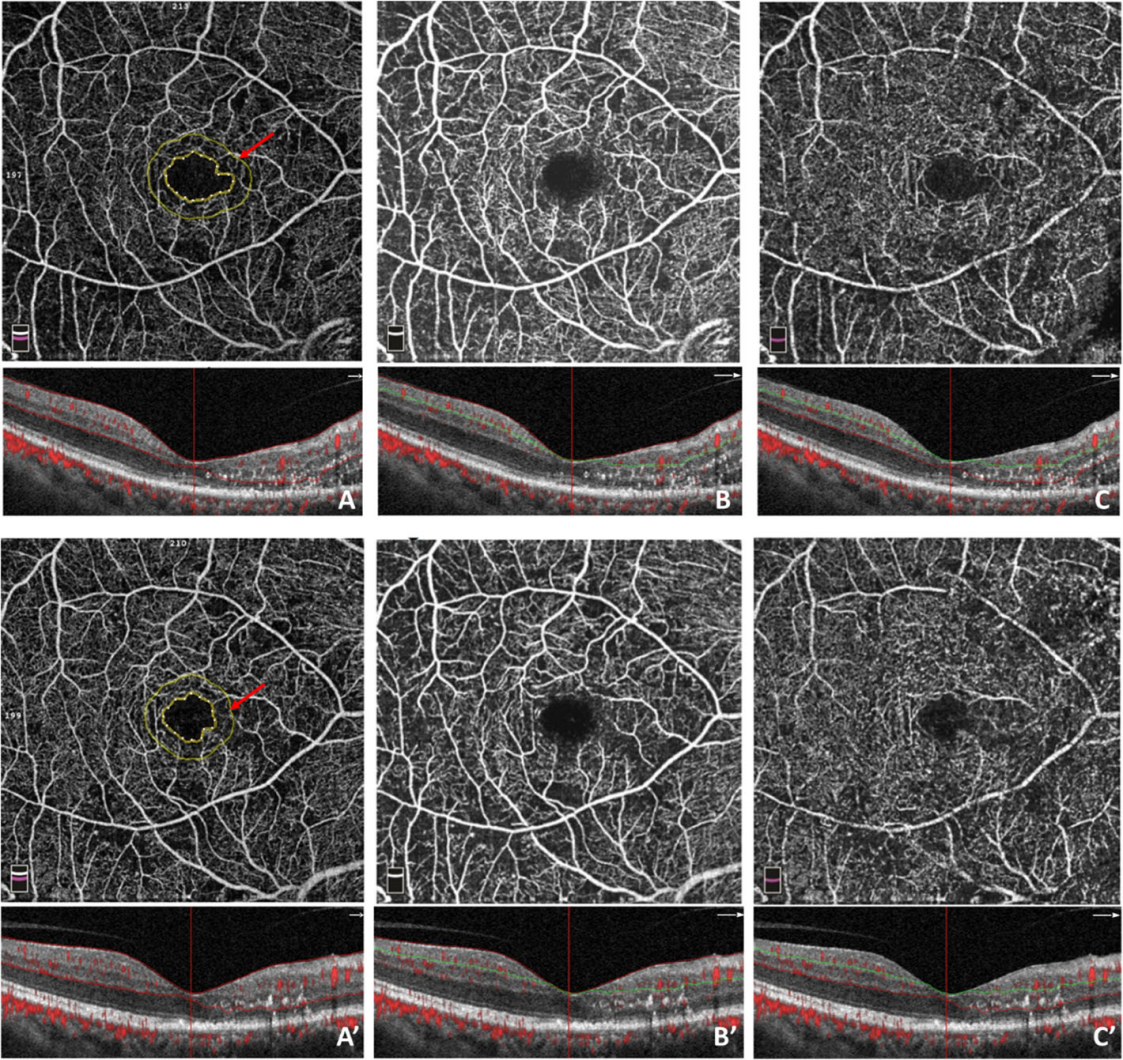
FAZ area clearly decreasing in the eye of a 59-year-old male, 6 months after receiving conbercept treatment. Notes: OCT angiography images (**a–c**) depict macular ischemia and apparent FAZ expansion captured in 6 × 6 mm2 sections in the macular area at month 0, Q-score = 8/10. **a** FAZ area with 0.604 mm2, showing severe FAZ area expansion and arch ring capillary network breakage; **b** Superficial capillary plexus (ILM-IPL), vessel density of 39.2%; **c** Deep capillary plexus (IPL-OPL), vessel density of 43.5%;. At month 6, OCT angiography images (**a’–c’**) showed FAZ area decrease, Q-score = 9/10. **a**’ FAZ area with 0.464 mm2; **b** Superficial capillary plexus vessel density of 40.4%; **c** Deep capillary plexus vessel density of 43.1%

After the patients were intraocularly injected with 0.5 mg of conbercept thrice, their BCVA improved and CFT decreased in both groups. The FAZ area of all patients decreased significantly at month 3 (*p* = 0.017), and remained stable until the month 6 (*p* = 0.311). Especially in the ischemic group, the FAZ area decreased from 0.510mm^2^ to 0.441mm^2^ (*p* = 0.003), continually decreasing to 0.427mm^2^ at the end point (*p* = 0.296). In contrast, the FAZ area did not expand significantly in the non-ischemic group. A representative case of DMI showed an apparent decrease in FAZ area, as illustrated in Fig. 2.

### Anti-VEFG therapy improved the vessel density in the macular region

**Fig. 3:**
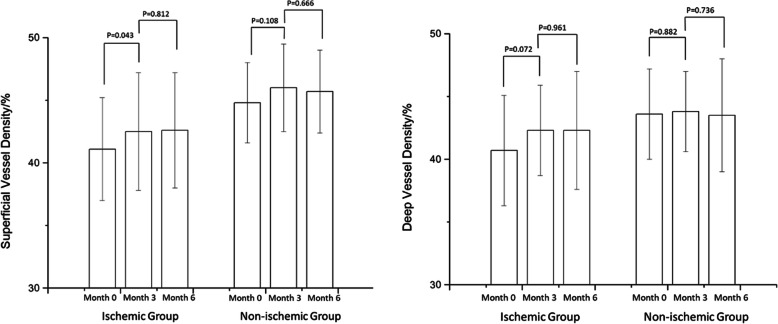
Changes of vessel density in both superficial plexus and deep plexus in all patients

**Fig. 4:**
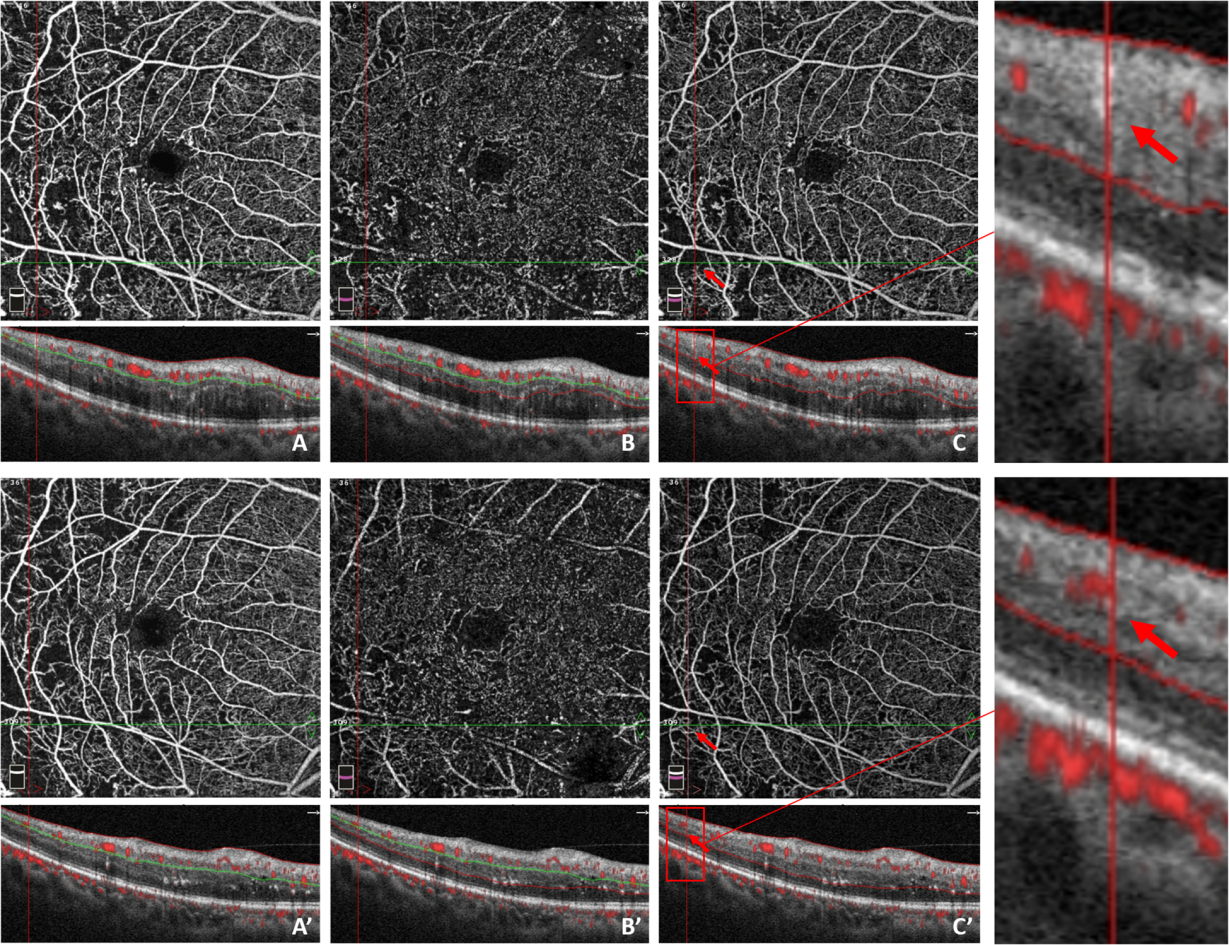
Reperfusion occurred in the right eye of a 53-year-old male, 6 months after receiving conbercept treatment. Red arrows and boxes indicate growth of new blood flow signal and reperfusion in the macular nonperfusion areas. OCT angiography images (**a–c**) at baseline illustrate the macular nonperfusion area, Q-score = 8/10. **a** Superficial capillary plexus, VD = 45.7%; **b** Deep capillary plexus, VD = 39.6; **c** Full retinal capillary plexus. OCT angiography images **a’–c’** at month 6 indicate reperfusion in previous macular nonperfusion areas, Q-score = 8/10. **a** Superficial capillary plexus, VD = 45.5%; **b** Deep capillary plexus, VD = 40.2%; **c** Full retinal capillary plexus

The superficial vessel density of all patients increased significantly at month 3 (p_month3_ = 0.008), and remained stable until the month 6 (p_month6_ = 0.944). The changes in the vessel density in both groups are depicted in Fig. 3. In the ischemic group, the superficial vessel density increased from 41.1% ± 4.1% to 42.5% ± 4.7% then to 42.6 ± 4.6% at the end point, and the deep vessel density changed from 40.7% ± 4.4% to 42.3 ± 3.6% then to 42.3% ± 4.7% at month 6. The capillary density increased in both superficial plexus (p_month3_ = 0.043) and deep plexus (p_month3_ = 0.072) in the first 3 months after anti-VEGF therapy, especially in the superficial plexus. Moreover, the vessel density remained stable during the observation period (p_month6_ = 0.812, p_month6_ = 0.961). In the non-ischemic group, the superficial vessel density changed from 44.8% ± 3.2% to 46.0% ± 3.5% then to 45.7 ± 3.3% (p_month3_ = 0.108, p_month6_ = 0.666), whereas the deep vessel density changed from 43.6 ± 3.6 to 43.8% ± 3.2% then to 43.5 ± 4.5% at the end point (p_month3_ = 0.882, p_month6_ = 0.736). Some patients had increased vessel density and reperfusion in previous nonperfusion area in the macular region (Fig. 4).

## Discussion

In our study, varying degrees of macular ischemia gained different therapeutic effects after anti-VEGF therapy in both the ischemic and non-ischemic groups. Furthermore, the macular perfusion status improved after the anti-VEGF therapy, especially in the FAZ area and superficial vessel density. The superficial vessel density is associated with DME development, whereas the vessel density in deep plexus corresponds to macular photoreceptors and is important to the oxygen requirements of photoreceptors and outer retina in patients with DMI. Some study found that increased number of capillary drop-out in superficial capillary plexus correlated with DR severity [24, 25]. Some patients with DMI obtained reperfusion at previous nonperfusion areas. As observed in Fig. 4, the OCTA detected that the FAZ area of a 59-year-old patient with DME, the structure of arch ring capillaries already disrupted, clearly decreased after receiving the anti-VEGF therapy and indicating rebuilding especially in the inferior-nasal macular fovea. Therefore, the anti-VEGF therapy improved macular ischemia and blood supply even the occurrence of macular reperfusion in some patients with DME.

Macular ischemia is a contraindication of anti-VEFG therapy, but some patients developed capillary nonperfusion after such therapy. Severe macular ischemia may be a limitation of visual acuity outcomes in patients with DME after receiving anti-VEGF therapy [26]. However, trials in DME suggested that anti-VEGF therapy did not induce retinal ischemia at least in healthy retina. In addition, repeated anti-VEGF therapy on macular perfusion in patients with DME did not cause treatment-related significant changes in FAZ sizes and capillary loss around the fovea. Patients with DME who had severe ischemia still achieved favorable changes in BCVA and central macular thickness after long-term anti-VEGF therapy [6, 7]; thus, anti-VEGF therapy may be an alternative for such patients. Therefore, patients with severe macular ischemia receiving anti-VEFG therapy should be individualized, and they need a comprehensive assessment of possible risks and closer follow-up to prevent the worsening of ischemia. Overall, patients with DMI in our study experienced improved eyesight and macular ischemia after anti-VEGF therapy.

Highly vitreous concentration of VEGF can lead to serious retina ischemia and hypoxia [27]. The retinal arterioles would contract immediately, and once ischemia occurs, vascular occlusion and RNP areas progression take place, resulting in retinal microvascular abnormality and neovascularization elsewhere, especially in patients with severe NPDR and PDR [28]. VEGF is the strongest angiogenic factor. The VEGF family in mammals mainly includes five species and PlGF. In DR, VEGF and PlGF disrupt the blood–retinal barrier, leading to DMI progression. Anti-VEFG therapy improves retinal hypoxia and ischemia by reducing the VEGF content. Conbercept is a new VEGFR fusion protein that reduces the concentration of VEGF and PlGF by specifically binding to VEGF-A, VEGF-B, and PlGF [29]. However, the molecular mechanism on how anti-VEGF therapy improves ischemia and realizes reperfusion remains unclear. A study using retinal ischemia animal models proved that anti-VEGF therapy could reduce autophagy and apoptosis rate and activate ischemia-damaged microglia to protect the retinal ganglion cells and bipolar cells [30]. In another study on tumor, VEGF inhibition can normalize peripheral cells, stabilize the basement membrane, remodel the immature vessels to a more mature version by destroying the vessels that lack peripheral cells, and provide frameworks for new vessels to grow in again by stabilizing the basement membrane. Surmising that the mechanism is the same in the retina [31, 32].

In this study, we adopted OCTA as the main evaluation method to observe the blood flow of the macular region and to quantify the macular vessel density. Despite acknowledging FA as a gold standard in the diagnosis of DR and classification of DMI, by comparing the FA and OCTA, we found that both tests were consistent in showing the vessels in the macular area, but OCTA is unacted on the leakage of fluorescein [14–16]. OCTA not only evaluated macular ischemia and DR severity but also predicted the peripheral nonperfusion by observing the change of FAZ size [33]. However, OCTA still has several defects. Fluid may induce segmentation artifacts, and OCTA quantitative metrics lacks consensus and normative database. And the main problem is that projection artifacts from large superficial vessels were inevitably included in the OCTA images of deep plexus, although we used PAR algorithm to reduce decorrelation tailing and projection artifact. This is a big challenge for us to measure the truly values of deep vessel density. But we believe that with the development of artifact removal algorithms, the measurements in vessel density might more precisely represent the actual flow especially in deep plexus. Other limitations in our study mainly include the small sample size and the short study period. Indeed, a part of studies used OCTA observing changes in macular perfusion and found there is no improvement in FAZ and vessel density after anti-VEGF [34–37]. The effects of anti-VEGF therapy on DMI need a larger sample size and a longer observation period to determine how anti-VEGF plays a role in improving macular ischemia and retinal ischemia and realizing reperfusion in nonperfusion areas.

## Conclusions

In our study, we observed the improvements of FAZ area and superficial vessel density in patients with DME after receiving intraocular injection of conbercept according to the 3+ PRN principle, especially greater in patients with DMI at baseline. Furthermore, conbercept had a positive impact on macular perfusion status and promoted reperfusion in macular nonperfusion areas. Lastly, OCTA may be used as an important imaging modality to evaluate retinal vasculature and to quantify the perfusion status in DR.

## Supplementary information

**Additional file 1:.**

## Data Availability

All data is included in Excel sheet, and medical records and picture information is included in this published article and supplementary files.

## Abbreviations

DR: Diabetic retinopathy
DMI: Diabetic macular ischemia
DME: Diabetic macular edema
FAZ: Foveal avascular zone
MNP: Macular nonperfusion
RNP: Retinal nonperfusion
VEGF: Vascular endothelial growth factor
VEGFR: Vascular endothelial growth factor receptor
PIGF: Placental growth factor
FA: Fluorescein angiography
OCT: Optical coherence tomography
OCTA: Optical coherence tomography angiography
CFT: Central fovea thickness
PRN: Pro re nata
SSADA: Split-spectrum amplitude decorrelation angiography
MCT: Motion correction technology
PAR: Projection artifact removal
BCVA: Best corrected visual acuity
ILM: Internal limiting membrane
IPL: Inner plexiform layer
OPL: Outer plexiform layer
NPDR: Non-proliferative diabetic retinopathy
PDR: Proliferative diabetic retinopathy
GHb: Glycated hemoglobin
